# The UCSC Interaction Browser: multidimensional data views in pathway context

**DOI:** 10.1093/nar/gkt473

**Published:** 2013-06-08

**Authors:** Christopher K. Wong, Charles J. Vaske, Sam Ng, J. Zachary Sanborn, Stephen C. Benz, David Haussler, Joshua M. Stuart

**Affiliations:** ^1^Biomolecular Engineering Department, University of California, Santa Cruz, 1156 High Street, Santa Cruz, CA 95064, ^2^Five3 Genomics, 101 Cooper St., Santa Cruz, CA 95060 and ^3^Howard Hughes Medical Institute, University of California, Santa Cruz. 1156 High Street, Santa Cruz, CA 95064, USA

## Abstract

High-throughput data sets such as genome-wide protein–protein interactions, protein–DNA interactions and gene expression data have been published for several model systems, especially for human cancer samples. The University of California, Santa Cruz (UCSC) Interaction Browser (http://sysbio.soe.ucsc.edu/nets) is an online tool for biologists to view high-throughput data sets simultaneously for the analysis of functional relationships between biological entities. Users can access several public interaction networks and functional genomics data sets through the portal as well as upload their own networks and data sets for analysis. Users can navigate through correlative relationships for focused sets of genes belonging to biological pathways using a standard web browser. Using a new visual modality called the CircleMap, multiple ‘omics’ data sets can be viewed simultaneously within the context of curated, predicted, directed and undirected regulatory interactions. The Interaction Browser provides an integrative viewing of biological networks based on the consensus of many observations about genes and their products, which may provide new insights about normal and disease processes not obvious from any isolated data set.

## INTRODUCTION

The behavior of a cell or cellular organism is orchestrated by networks of interacting genes. Large-scale projects such as ENCODE and the Cancer Genome Atlas (TCGA) are underway to uncover the genomic, epigenomic and functional genomic landscapes of many different cells. As high-throughput techniques such as DNA and RNA sequencing become even more efficient, a key challenge lies in developing integration and visualization approaches to shed light on the significant pathways underlying intrinsic, adaptive and developmentally programmed cellular changes that can be aberrantly regulated in disease processes.

Heatmaps are valuable tools aiding the human eye to detect patterns of activity associated with subsets of genes over subsets of samples, introduced in bioinformatics by the seminal work of Weinstein *et al.* (1) and later Eisen *et al.* (2). Gene set enrichment analysis tools such as a Fisher’s exact overlap test or the use of gene set enrichment analysis (3) can be used to determine statistically significant pathways overrepresented in clusters of genes. At this point in the analysis, viewing the data in the context of an enriched pathway may provide significant interpretative power. Using a pathway-guided view, correlations between genomic and transcriptional events could be traced through the regulatory logic of interacting genes, the expression of genes in common regulatory programs could be verified, the consequences of copy number changes on the transcriptome could be mapped; the RNA expression of microRNAs against the RNA or protein-level expression of predicted targets could help identify those targets under active silencing control, and so on.

While many tools for viewing genetic networks are available such as Cytoscape, Reactome, STRING, IMP and WikiPathways (4–13), few provide a solution that allows a biologist to interpret a pathway’s activity deduced from multiple platforms of data. It is of paramount importance that new tools enter this space that go beyond network ‘hairball’ viewing that can both reduce the information overload of massive data sets, while at the same time focus attention on critically important pathways and trends as observed in collected data. We have developed an interactive browsing tool for viewing multiple measures of gene activity such as gene expression, copy number, methylation, and somatic mutation data in the context of cellular pathways. The pathways may include any number of different ‘features’ (proteins, complexes, small molecules and cellular processes) connected by sets of known regulatory interactions. This new tool should aid researchers in viewing both publicly available as well as user-defined data sets and pathway collections.

## DESCRIPTION OF THE INTERACTION BROWSER

### Overview

**Figure 1.**
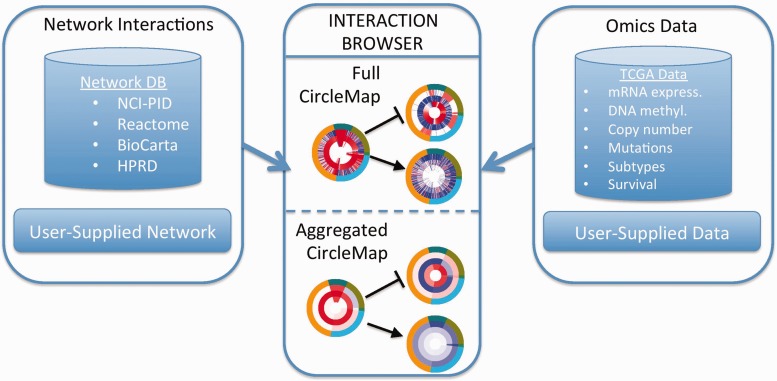
Overview of the UCSC IB workflow. Users select or supply (i) a network of interactions (left box) and (ii) a data set for viewing (right box). The data are viewed for a selected set of genes in the main panel using a CircleMap display (center box). Data for individual samples are displayed as individual ‘tick marks’, different data platforms are displayed as separate rings (‘Full CircleMap’). Samples can be aggregated together into groups, displayed as segments with averaged color hue (‘Aggregated CircleMap’).

We propose a solution called the University of California, Santa Cruz (UCSC) Interaction Browser (IB) that provides several features for viewing biological data sets overlaid on genetic pathways. The IB is a web portal to explore evidence pertaining to the regulatory interactions present in a genetic pathway (Figure 1). The browser takes two main inputs: (i) a network of interactions among gene products and (ii) ‘omics’ data measuring evidence on multiple genes across multiple samples.

Interaction data sets can be selected from a backend database of networks or supplied by the user. A genetic network is displayed as a graph with nodes and edges. A user can select a set of genes representing a pathway of interest and a set of networks to explore known functional interactions documented among the selected genes. Molecular entities such as genes, gene products, complexes, families, small molecules and abstract cellular processes can be rendered. Genes are keyed based on HUGO gene symbols to facilitate the communication of protein-level information across a variety of functional networks, genetic pathways, as well as measurement platforms such as microarrays, RNA-seq, SNP-chip copy number and proteomics measurements.

Currently, the IB contains 929 curated pathways available for viewing including those ingested from Reactome (6), Biocarta (http://www.biocarta.com/) and NCI-PID (8) (Supplemental Table S1). In addition, the IB currently contains 59 networks predicted from functional genomics sources such as those derived from protein interaction databases including BioGRID (14) (19 networks) or extracted from the meta-analysis of many gene expression data sets to infer co-expression networks (Supplemental Table S2). The IB provides different visual styles to display multiple types of linkages in networks or pathways. Directed edges allow viewing curated regulatory and signaling interactions. Arrowheads are used for activating actions and lines ending with a small perpendicular line segment (‘T-bars’) are used for inhibitory actions.

The user interface (UI) is geared toward the display of smaller focused pathways, for example, those pathways containing 10–100 genes. However, the IB does provide access to much larger networks that can be drawn on. For example, the UCSC Superimposed Pathway (‘SuperPathway’) combines the interactions collected from NCI-PID, BioCarta and Reactome into one network currently containing 32 000 directed and undirected interactions among over 7000 proteins (15). These larger pathways can be loaded into the background, allowing smaller subnetworks of interest to be brought into the display using filtering steps. This allows users to query the background network while avoiding the display of massively complex interaction sets that can tax browser responsiveness.

To enable identifying high-confidence relations, multiple interaction networks can be used for filtering or display simultaneously. Borrowing a convention from the UCSC Genome Browser (16), different networks of interactions are available as overlays onto a set of genes as separate network ‘tracks’. If an interaction between two genes in the current display is present in a selected track, it is rendered using colors that distinguish the database source of the interaction. In this way, several network tracks can be visualized at once to investigate the degree to which any functional linkage between genes is supported by multiple data sets and platforms, thereby raising the confidence level of particular links. For example, regulatory interactions from transcription factors (TFs) to targets (directed) can be displayed alongside physical protein–protein interactions (undirected) detected from yeast two-hybrid or co-immunoprecipitation assays. In this context, the protein–protein links between TFs can help identify putative transcription factor complexes that share common targets, which in turn may be connected by co-expression–derived linkages (undirected).

The IB provides a portal to visualize publicly available genomics data sets, functional genomics data sets, phenotype data on samples and outcome data on patients. Currently, 22 cancer genomics data sets from TCGA are available, where each contains multiple different measurement platforms including copy number abnormalities (CNA), DNA methylation patterns, gene expression levels and protein levels from reverse phase protein arrays. Data summarized at the gene level (Level 3 data) as well as associated clinical information on patient outcomes and relevant subtypes are obtained from the TCGA Data Repository (http://tcga-data.nci.nih.gov/tcga/). TCGA data are also ingested from the Broad Institute’s Firehose pipeline (http://www.broadinstitute.org/cancer/cga/Firehose) that provides higher levels of interpretive results such as significantly mutated genes, focal regions of amplification from GISTIC (17) analysis of CNA and pathway-level inferences from the UCSC-developed PAthway Recognition Algorithm using Data Integration on Genomic Models (PARADIGM) engine. The IB is particularly well suited for viewing inferences from PARADIGM’s integrative analysis. Briefly, PARADIGM was developed to infer the activities of genes in the context of pathways by integrating any number of functional genomic data sets in a patient sample (18). Other cancer genomics data sets are also available through the IB including several breast cancer studies, several lung, blood, skin, brain, ovarian, pancreatic cancer tracks, two COSMIC (19) tracks, NCI60 cell line data, the Connectivity Map (20) data sets, and the Cell Line Encyclopedia (21) data measuring expression and drug response on different cancer cell lines. A description of the collection can be found in Supplemental Table S3.

Users can upload their own data matrices to the IB for viewing. The input file format is a matrix of scores in which the rows represent the data for a gene and the columns represent the data for samples. The first column identifies the gene, preferably the HUGO name. The first row lists the column names, usually pertaining to unique sample identifiers for each column. Once the data matrix has been uploaded, a custom checkbox can be used to select the data set that is private only to the user’s current IB session. The IB also allows users to provide their own files for gene sets and networks. The IB webpage has information about how this API may be accessed programmatically to query the IB data sets. CircleMaps are generated through an HTTP GET mechanism. Open source Python scripts are available for free from the IB website for users to generate their own CircleMaps outside of the IB, which may be more convenient for users to generate CircleMaps of their own data set files.

The IB’s website gives biologists immediate access to all of the networks and omics data without software installation for a variety of modern web browsers. The UI is developed with the Google Web Toolkit (GWT), building on Asynchronous JavaScript and XML (AJAX) created using Java development tools. The network-drawing portion of the IB is implemented in Scalable Vector Graphics (SVG). The AJAX UI treats the SVG as an object in the Document Object Model tree, which may be dynamically modified. The backend of the IB uses Apache Tomcat to serve up the GWT-compiled JavaScript UI as well as Java servlets for accessing and processing data. A MySQL server is used as the data source.

### CircleMaps: dynamic, coordinated viewing

Pathways and networks can enrich the viewing of high-throughput data sets by focusing the exploration of data on established or predicted gene regulatory logic. One of the main IB features is the introduction of the ‘CircleMap’ concept for viewing omics data sets. A CircleMap displays multiple data sets as nested rings pivoted around each protein. Each ring represents measurements of a gene property across any number of samples. Rings are composed of a series of colored ‘spokes’, where each spoke represents one sample in the data set (e.g. cell line or tumor sample). All of the data for a particular sample, oriented for a particular gene, can be viewed as one radiating spoke. Importantly, CircleMaps for multiple genes are coordinated so that a sample is located at the same angular position, allowing the results to be easily traced across an entire pathway.

**Figure 2.**
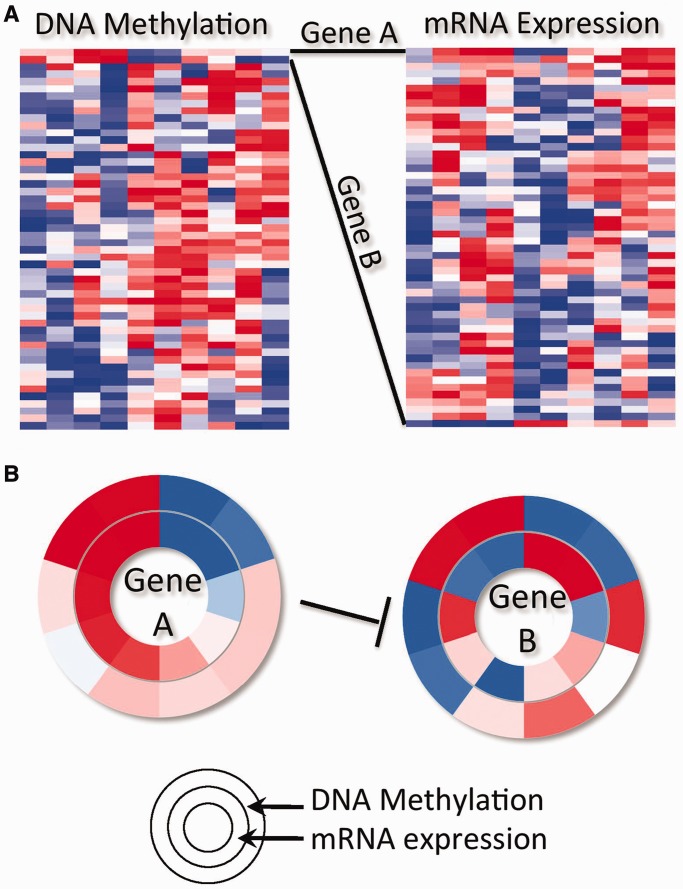
A toy example illustrating relationship of a CircleMap to a standard heatmap. (**A**) Left matrix represents DNA methylation data; right matrix mRNA expression data for the same genes (rows) and 10 samples (columns) as for the DNA methylation data. Two genes (gene A and gene B) co-cluster in the DNA methylation data (top) but do not in the mRNA data owing to gene B’s anti-correlation with gene A’s expression. (**B**) CircleMap shows data across the 10 samples for genes A and B with mRNA expression on the inner ring and DNA methylation on the outer ring. Each ‘spoke’ represents one sample (column) in the heatmap of part A.

Figure 2 illustrates the comparison of a CircleMap to a heatmap to illustrate the complementary strength added by the CircleMap. The heatmaps for two theoretical data sets on the same samples (columns) are shown, one containing DNA methylation levels for CpG islands near the promoters of a set of genes (left matrix) and another containing gene expression levels for the same set of genes (right matrix). Such multidimensional data sets are becoming common especially from national and international consortia. The two-dimensional clustered matrix display of the heatmaps allows one to visually see the dominant patterns in the data. For example, most genes have increasing DNA methylation and decreasing mRNA expression (left to right orientation in the heatmap), an anti-correlation relationship that is expected because DNA methylation tends to silence gene promoters. However, the heatmap view can overlook specific relations between a subset of interacting genes. In this contrived example, gene A’s product inhibits the expression of gene B. While the DNA methylation profiles of the genes are positively correlated, leading to the co-clustering of genes A and B, the mRNA expression of the two are anti-correlated and therefore gene B is sorted far from gene A in the mRNA heatmap. Identifying the presence of this confirmed regulatory relationship is therefore problematic in the heatmap because a user’s eye has to relate one or a few gene vectors, embedded in a much larger collection, to other vectors across long visual distances. On the other hand, the CircleMap view readily reveals this kind of relationship using a single sort of the samples based on gene A’s mRNA expression levels.

Several fundamental CircleMap operations including spoke coordination, spoke sorting and spoke aggregation provide simple but powerful view modalities. First, all of the nested rings in the display are coordinated such that every gene maintains the same spoke order for the samples. Second, by selecting any ring, the spokes are sorted in ascending or descending order according to the data values of the ring and this order is propagated to all other rings. Third, samples can be grouped together by disease subtypes, tissues, common phenotypes and patient outcomes. All of the values within a group can be averaged together and displayed as a ring segment. The combination of these operations allows the eye to pick up trends and detect correlations between particular genes that exist within important subsets.

## CASE STUDY: ANALYSIS OF A TCGA CANCER GENOMICS DATA SET

**Figure 3.**
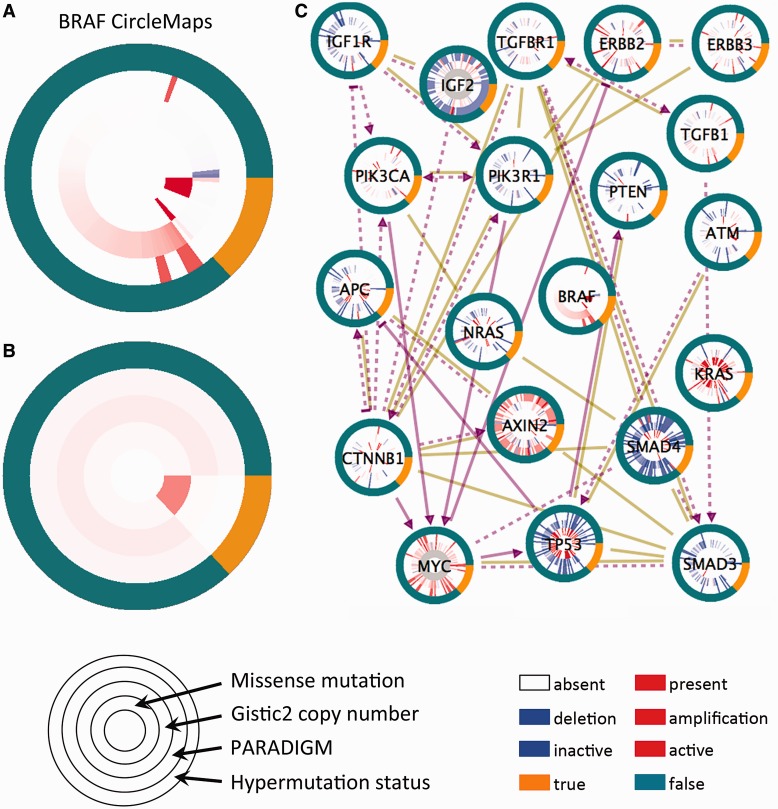
Case study of the TCGA colorectal adenocarcinoma data set. (**A**) A zoom-in view of the BRAF oncogene’s CircleMap showing the full detail of all samples as individual spokes. Rings correspond to (inside to outside) somatic missense mutations, copy number estimates from the GISTIC algorithm, PARADIGM pathway inferences and a hypermutation phenotype indicator. (**B**) Same as in part **A** but the values within the hypermutated and non-hypermutated groups are averaged within each ring showing the aggregated view. (**C**) CircleMap display of most of the Wnt-, TGF-beta and PI3K-signaling members regulating MYC oncogene activation. Both curated regulatory links from the UCSC SuperPathway are shown (purple directed links) as well as protein–protein interactions collected from human protein reference database (brown undirected links). All nodes are sorted according to the hypermutated versus non-hypermutated as the primary sort and BRAF mutation as the secondary sort, controllable from the zoom-in on BRAF.

To illustrate the functionality of the IB, we analyzed the TCGA colorectal carcinoma (CRC) data set (22). In CRC, the MYC oncogene is often activated through disruption of normal signaling of the Wnt or TGF-beta pathways, accounting for >90% of patient tumors. For example, APC mutations lead to the loss of inhibition of MYC by beta-catenin (CTNNB1). However, a minority of CRC patients have an elevated number of mutations in their exomes (>200) and are associated with disruptions in different signaling pathways, one of which is disruption of the RAS-MAPK and PI3K signaling pathways.

v-raf murine sarcoma viral oncogene homolog B1 (BRAF) is preferentially mutated in a subset of CRC patients’ tumors harboring highly elevated rates of somatic mutations (>200 mutations in the exome), known as the ‘hypermutated’ class of tumors. Inspection of BRAF’s CircleMap easily reveals this known phenomenon (Figure 3A). In addition, the figure also shows gene expression levels and CNA rings selected for the BRAF gene. By sorting both the hypermutated subtype ring and the BRAF mutation ring, the enrichment of BRAF mutations in the hypermutated patients becomes apparent. The trend can be confirmed visually by converting the view to Figure 3B, which displays the average values of the rings within each of the patient subgroups.

The IB allows a researcher to load a moderately sized pathway diagram for CircleMap viewing. Figure 3C shows most of the genes in the CRC pathway discussed in the TCGA marker paper (22), showing many of the components involved in the Wnt, TGF-beta and PI3K signaling pathways impinging on MYC oncogene regulation. The visualization allows a large amount of the TCGA data to be quickly screened by eye. Experimentally determined levels, such as the CNA and mutation values for all of the genes, can be viewed alongside inferred activities from a pathway analysis method such as PARADIGM. The data can be sorted and grouped based on the hypermutation versus non-hypermutated dichotomization to allow viewing the data along familiar patient subtypes.

## SUMMARY AND FUTURE DIRECTIONS

The visualization approach described here is complementary to standard heatmap viewing. Ideally, a system could connect both views together so that a user would be able to switch between the two as analysis dictates. The UCSC Cancer Genomics Browser (https://genome-cancer.ucsc.edu/) is a set of web-based tools to display, investigate and analyze cancer genomics data and its associated clinical information (23). The browser uses dynamic heatmaps to navigate cohorts of samples, allowing users to correlate genomic or proteomic data with phenotypic information. In the future, we plan to develop a tight interconnection of the IB with the Cancer Genomics Browser.

The IB interactively displays multiple data sets overlaid onto a genetic pathway using an intuitive CircleMap. The technique allows various different platforms of data to be stacked concentrically around genes of interest. The network interaction databases are updated every 6 months, and cancer genomics data sets are updated as they are made available from projects like TCGA. Future versions will include even more interaction types and extend to additional platforms of data such as microRNAs and Chip-Seq data. The Clinical Proteomic Tumor Analysis Consortium (24) and Library of Integrated Network-Based Cellular Signatures (25) projects are currently producing massive amounts of proteomics data on tumor samples and cell lines. These data sets often focus on subsets of genes, measuring their response under thousands of different combinations of perturbations such as siRNA knockdowns or treatments with multiple molecular compounds. Experiments will need to be grouped by perturbagen, cell line and other experimental parameters. The IB will provide a useful alternative mechanism for viewing such ‘lopsided’ data sets that cover only a few genes in a targeted pathway but over many conditions.

Finally, we have described an approach that is necessarily gene-centric in which the data are wrapped around gene products and the interactions depict known (or predicted) gene–gene relations. One could use the same CircleMap and interaction metaphor to provide *sample-centric* views of the data sets. This view would wrap columns from a heatmap around individual samples rather than rows around genes as is discussed here. Ticks in the sample-centric view could correspond to genes and therefore be sorted or grouped by their membership in known pathways or functional modules (analogous to the grouping by patient subtypes). Samples could be connected to one another based on shared properties such as the presence of a common mutation (e.g. TP53 or PIK3CA) or based on the similarity in clinical or other phenotypic outcomes. Directed graphs among patient samples could represent censored survival relations. The sample-centric view may be worth investigating as a way to browse genomics and proteomics data sets for precision medicine applications. In conclusion, the IB represents a flexible new tool for coordinated viewing of rich multimodal data sets to discover data trends present among genes that participate in pathways of critical cellular functions.

## SUPPLEMENTARY DATA

Supplementary Data are available at NAR Online: Supplementary Tables 1–3.

## FUNDING

National Institutes of Health LINCS program (to J.M.S and C.W.); National Cancer Institute [5U24CA143858]; an National Science Foundation (NSF) CAREER award (to J.M.S.); Howard Hughes Medical Institute (to D.H.). Funding for open access charge: NSF CAREER award.

*Conflict of interest statement.* None declared.
